# Supplementary material for: Linear parameter-varying model for a refuellable zinc–air battery
Source: R Soc Open Sci. 2020 Dec 9;7(12):201107. doi: 10.1098/rsos.201107 (PMC7813229; doi:10.1098/rsos.201107)
Supplement: Supplementary figures and tables [file rsos201107supp2.docx]

Linear Parameter Varying Model for a Refuellable Zinc-air Battery

Woranunt Lao-atiman^1^, Sorin Olaru^2^, Sette Diop^2^, Sigurd Skogestad^3^, Amornchai Arpornwichanop^1,4^, Rongrong Cheacharoen^5^ , Soorathep Kheawhom^1,4,*^

^1^ Department of Chemical Engineering, Faculty of Engineering, Chulalongkorn University, Thailand

^2^ Université Paris-Saclay, CNRS, CentraleSupélec, Laboratoire des signaux et systèmes, Gif sur Yvette, France

^3^ Department of Chemical Engineering, Norwegian University of Science and Technology, Trondheim, Norway

^4^ Center of Excellence in Process and Energy Systems Engineering, Chulalongkorn University, Bangkok, Thailand

^5^ Metallurgy and Materials Science Research Institute, Chulalongkorn University, Bangkok, Thailand

**Table S1.** The location of the data used in this work.

| Data name | Data location | |
| --- | --- | --- |
|  | Data file^1,2^ | Sheet [time] |
| 0T100A | StepDischarge.xlsx | 100STEP0-100-0 [1 s – 302 s] |
| 0T100B | StepDischarge.xlsx | 100STEP0-100-0 [585 s – 911 s] |
| 0T100C | StepDischarge.xlsx | 100STEP0-100-0 [1185 s – 1502 s] |
| 100T0A | StepDischarge.xlsx | 100STEP0-100-0 [291 s – 598 s] |
| 100T0B | StepDischarge.xlsx | 100STEP0-100-0 [903 s – 1195 s] |
| 100T0C | StepDischarge.xlsx | 100STEP0-100-0 [1491 s – 1801 s] |
| 0T450A | StepDischarge.xlsx | 450STEP0-450-0 [1 s - 303 s] |
| 0T450B | StepDischarge.xlsx | 450STEP0-450-0 [588 s - 899 s] |
| 0T450C | StepDischarge.xlsx | 450STEP0-450-0 [1181 s – 1499 s] |
| 450T0A | StepDischarge.xlsx | 450STEP0-450-0 [292 s – 598 s] |
| 450T0B | StepDischarge.xlsx | 450STEP0-450-0 [890 s – 1197 s] |
| 450T0C | StepDischarge.xlsx | 450STEP0-450-0 [1491 s – 1801 s] |
| 0T900A | StepDischarge.xlsx | 900STEP0-900-0 [1 s - 313 s] |
| 0T900B | StepDischarge.xlsx | 900STEP0-900-0 [590 s - 901 s] |
| 0T900C | StepDischarge.xlsx | 900STEP0-900-0 [1180 s – 1502 s] |
| 900T0A | StepDischarge.xlsx | 900STEP0-900-0 [301 s – 601 s] |
| 900T0B | StepDischarge.xlsx | 900STEP0-900-0 [889 s – 1193 s] |
| 900T0C | StepDischarge.xlsx | 900STEP0-900-0 [1491 s – 1813 s] |
| 400T500R | StepDischarge.xlsx | 100STEP400-500 |
| 500T1000R | StepDischarge.xlsx | 500STEP500-1000-500 |
| MULTI | Suplementary.xlsx | MULTI |
| VARIOUS | Suplementary.xlsx | VARIOUS |

^1^ ’StepDischarge.xlsx’ is located in previous published paper [1].; ^2^ ’Supplementary.xlsx’ is located in supplementary file.

**Table S2.** Parameter values of linear state space models

| Model name | Identification data | *A* | *B* | *C* | *D* | Fit % ^1^ |
| --- | --- | --- | --- | --- | --- | --- |
| SS0T100A | 0T100A | 0.8880 | 0.6476 | 0.3350 | 0.1288 | 97.0686 |
| SS0T100B | 0T100B | 0.8757 | -0.5743 | -0.3608 | 0.4943 | 98.2905 |
| SS0T100C | 0T100C | 0.8771 | -0.5085 | -0.4326 | 0.4338 | 98.1266 |
| SS100T0A | 100T0A | 0.9503 | -0.1232 | -0.6526 | 0.4654 | 97.6896 |
| SS100T0B | 100T0B | 0.9567 | 0.0971 | 0.7602 | 0.4655 | 97.6354 |
| SS100T0C | 100T0C | 0.9583 | 0.0950 | 0.7492 | 0.5061 | 97.6246 |
| SS0T450A | 0T450A | 0.6433 | -0.5052 | -0.3498 | 0.5296 | 97.1758 |
| SS0T450B | 0T450B | 0.7362 | 0.2783 | 0.5663 | 0.4717 | 98.9290 |
| SS0T450C | 0T450C | 0.7421 | 0.2121 | 0.8703 | 0.3710 | 98.6913 |
| SS450T0A | 450T0A | 0.9132 | -0.0505 | -1.1848 | 0.3504 | 98.2935 |
| SS450T0B | 450T0B | 0.9349 | -0.0357 | -1.2178 | 0.4012 | 98.2564 |
| SS450T0C | 450T0C | 0.9421 | -0.0313 | -1.2382 | 0.4172 | 98.3725 |
| SS0T900A | 0T900A | 0.7885 | -0.1840 | -0.1394 | 0.6378 | 97.5129 |
| SS0T900B | 0T900B | 0.6926 | 0.1188 | 0.9817 | 0.3954 | 98.5239 |
| SS0T900C | 0T900C | 0.7572 | 0.0947 | 0.9458 | 0.4209 | 98.0704 |
| SS900T0A | 900T0A | 0.9251 | -0.0220 | -1.2967 | 0.3702 | 98.4046 |
| SS900T0B | 900T0B | 0.9426 | -0.0166 | -1.4480 | 0.3545 | 98.2246 |
| SS900T0C | 900T0C | 0.9208 | 0.0258 | 1.4074 | 0.3190 | 98.1882 |

^1^ Fit % indicates how well the model prediction fits the estimation data and expressed as: $\text{100}\left( \text{1-}\frac{\left\| \text{y-}\hat{\text{y}} \right\|}{\left\| \text{y-mean(y)} \right\|} \right)$.

**Table S3.** Parameter values of nonlinear Hammerstein-Wiener model

| Model name | nonlinear A | nonlinear B |
| --- | --- | --- |
| Identification data | MULTI | VARIOUS |
| *P_1_* ^1^ | 0.8617 | 1.9557 |
| *P_2_* ^1^ | -1.4124 | -2.8723 |
| *P_3_* ^1^ | 0.9046 | 1.6107 |
| *P_4_* ^1^ | 0.0180 | 0.0170 |
| *b_0_* ^2^ | 1.0000 | 1.0000 |
| *b_1_* ^2^ | -0.5570 | -0.9522 |
| *f_1_* ^3^ | -0.7979 | -0.9659 |
| Fit % ^4^ | 91.9066 | 91.1311 |

^1^ 3^rd^ degree polynomial: $\text{w}\text{ = }\mu_{\text{1}}\text{u}^{\text{3}}\text{ + }\mu_{\text{2}}\text{u}^{2}\text{ + }\mu_{\text{3}}\text{u +}\mu_{\text{4}}$; ^2^ $\text{B(z) = }\text{b}_{\text{0}}\text{ + }\text{b}_{\text{1}}\text{z}^{\text{-1}}\text{ for }\text{n}_{\text{b}}\text{ = }\text{2}$; ^3^ $\text{F}(z)\text{ = 1 + }\text{f}_{\text{1}}\text{z}^{\text{-1}}\text{ for }\text{n}_{\text{f}}\text{ = }\text{1}$;
^4^ Fit % indicates how well the model prediction fits the estimation data and expressed as: $\text{100}\left( \text{1-}\frac{\left\| \text{y-}\hat{\text{y}} \right\|}{\left\| \text{y-mean(y)} \right\|} \right)$

**Table S4.** Correlations for parameters of LPV model

| Parameter | Fitting correlation | Correlation Parameters |
| --- | --- | --- |
| *A* | 2^nd^ degree polynomial^1^ | *μ_1_* = 0.6464, *μ _2_* = -0.7996, *μ _3_* = 0.9411 |
| *B* | 2^nd^ degree polynomial | *μ _1_* = 0.1975, *μ _2_* = -0.1226, *μ _3_* = -0.0308 |
| *C* | 2^nd^ degree polynomial | *μ _1_* = -1.535, *μ _2_* = 2.533, *μ _3_* = -0.4454 |
| *D* | Linear function^2^ | *μ _1_* = 0.1049, *μ _2_* = 0.3931 |
| *BC* | Two-term exponential^3^ | *α* = 0.3992, *β* = -1.824, *γ* = -0.3485, *δ* = -10.84 |

^1^ 2^nd^ degree Polynomial: $\text{ξ}\text{ = }\mu_{\text{1}}\text{p}^{\text{2}}\text{ + }\mu_{\text{2}}\text{p + }\mu_{\text{3}}$; ^2^ Linear function: $\text{ξ}\text{ = }\mu_{\text{1}}\text{p}\text{ + }\mu_{\text{2}}$;
^3^ Two-term Exponential: $\text{ξ}\text{=}\text{ }\text{α}\text{exp(}\text{β}\text{p}\text{)}\text{ }\text{+}\text{ }\text{γ}\text{exp(}\text{δ}\text{p}\text{)}\text{)}$

**Table S5.** Fit % values^1^ of model predictions relating to Figure 4, 6 and 7

| Model name | Validation data | | | | | |
| --- | --- | --- | --- | --- | --- | --- |
|  | 0T100A | 0T900A | 400T500R | 500T1000R | MULTI | VARIOUS |
| SS0T100A | 97.0686 | -434.8147 | -1343.1925 | -656.3209 | -107.5684 | -8.0848 |
| SS0T450A | -97.7554 | -29.5903 | 19.5515 | -73.4169 | 49.7958 | 48.6385 |
| SS0T900A | -157.3168 | 97.5129 | -344.9635 | 37.0096 | 66.1462 | 21.0252 |
| LPV | 86.2316 | 85.8450 | 49.6094 | 65.3644 | 89.7656 | 86.8607 |
| Nonlinear A | 60.9576 | 85.9172 | 50.5309 | 56.9882 | 91.9066 | 87.4859 |
| Nonlinear B | 68.5313 | 53.6687 | 41.6458 | -22.4831 | 82.3515 | 91.1311 |

^1^ Fit % indicates how well the model prediction fits the estimation data and expressed as: $\text{100}\left( \text{1-}\frac{\left\| \text{y-}\hat{\text{y}} \right\|}{\left\| \text{y-mean(y)} \right\|} \right)$.


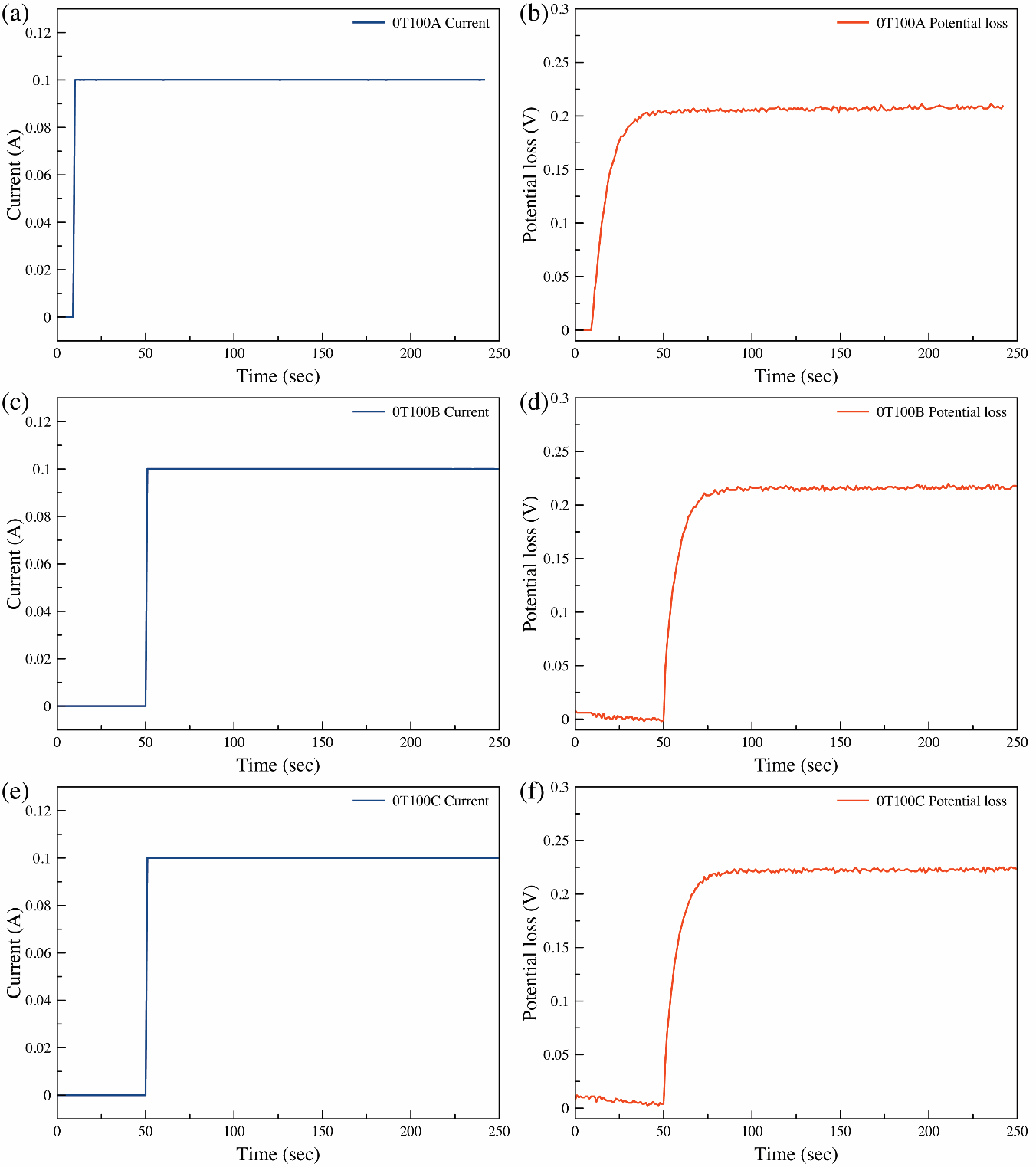


**Figure S1.** Response signals for data 0T100 including (a,c,e) current and (b,d,f) potential loss vs time.


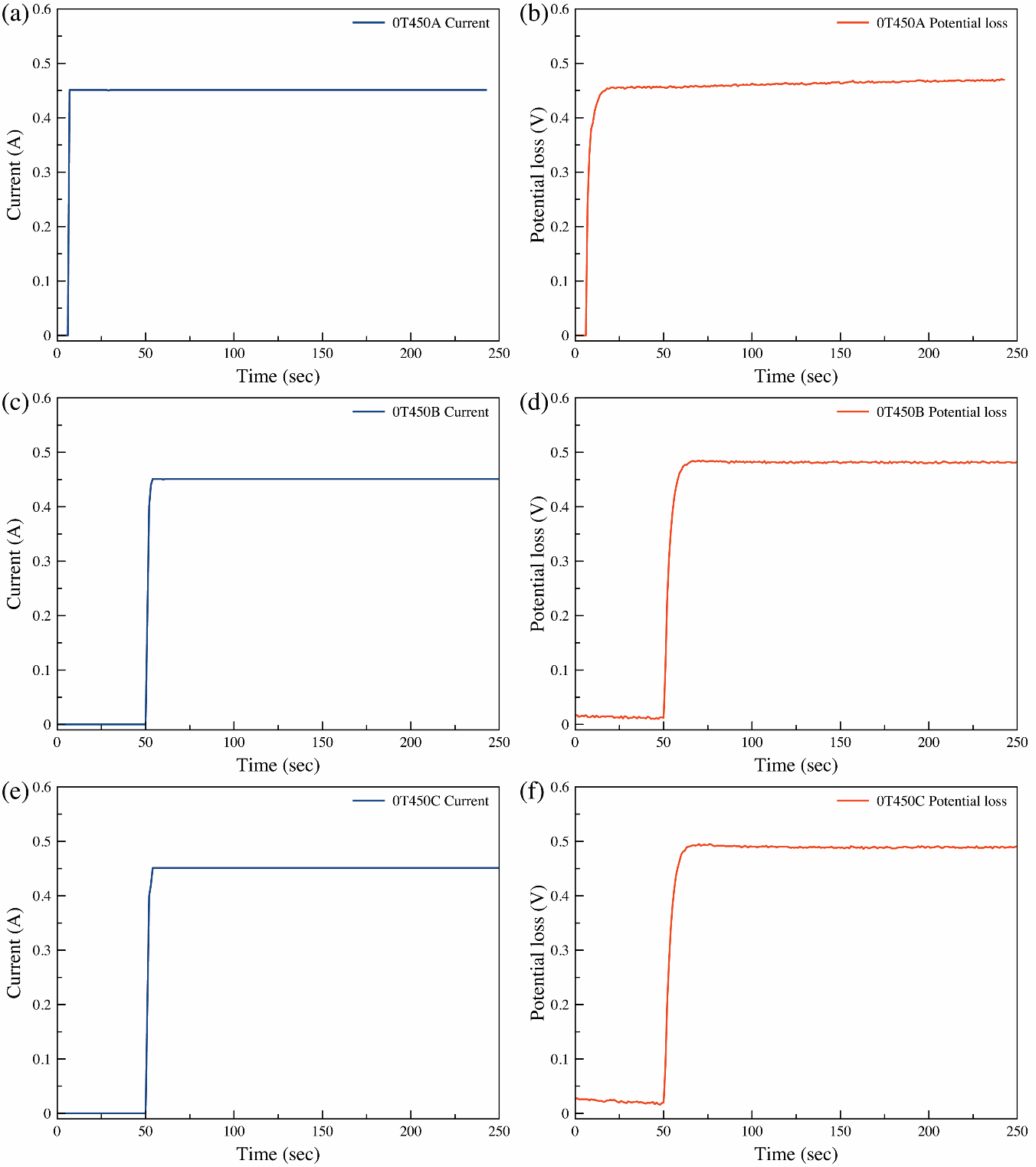


**Figure S2.** Response signals for data 0T450 including (a,c,e) current and (b,d,f) potential loss vs time.


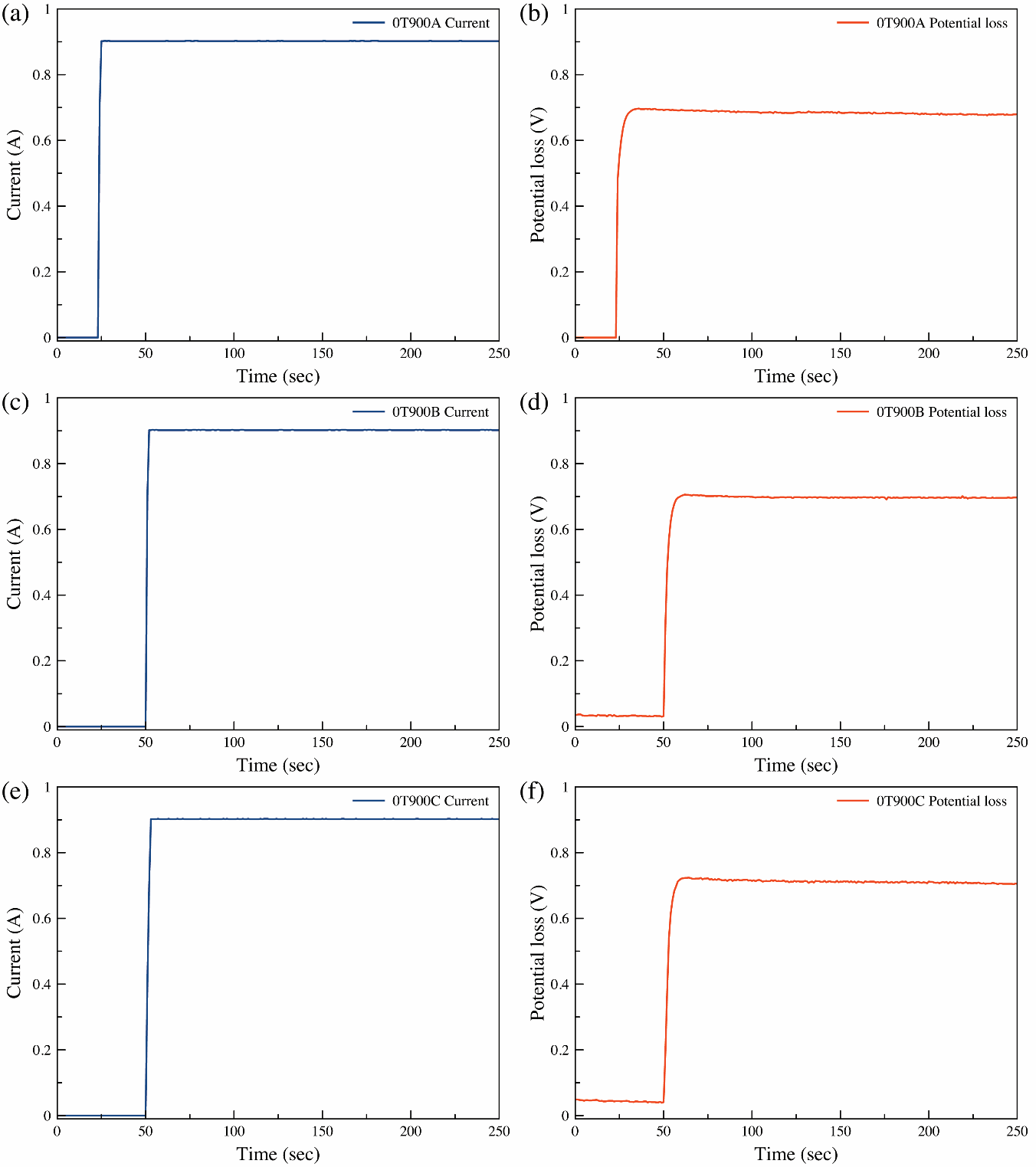


**Figure S3.** Response signals for data 0T900 including (a,c,e) current and (b,d,f) potential loss vs time.


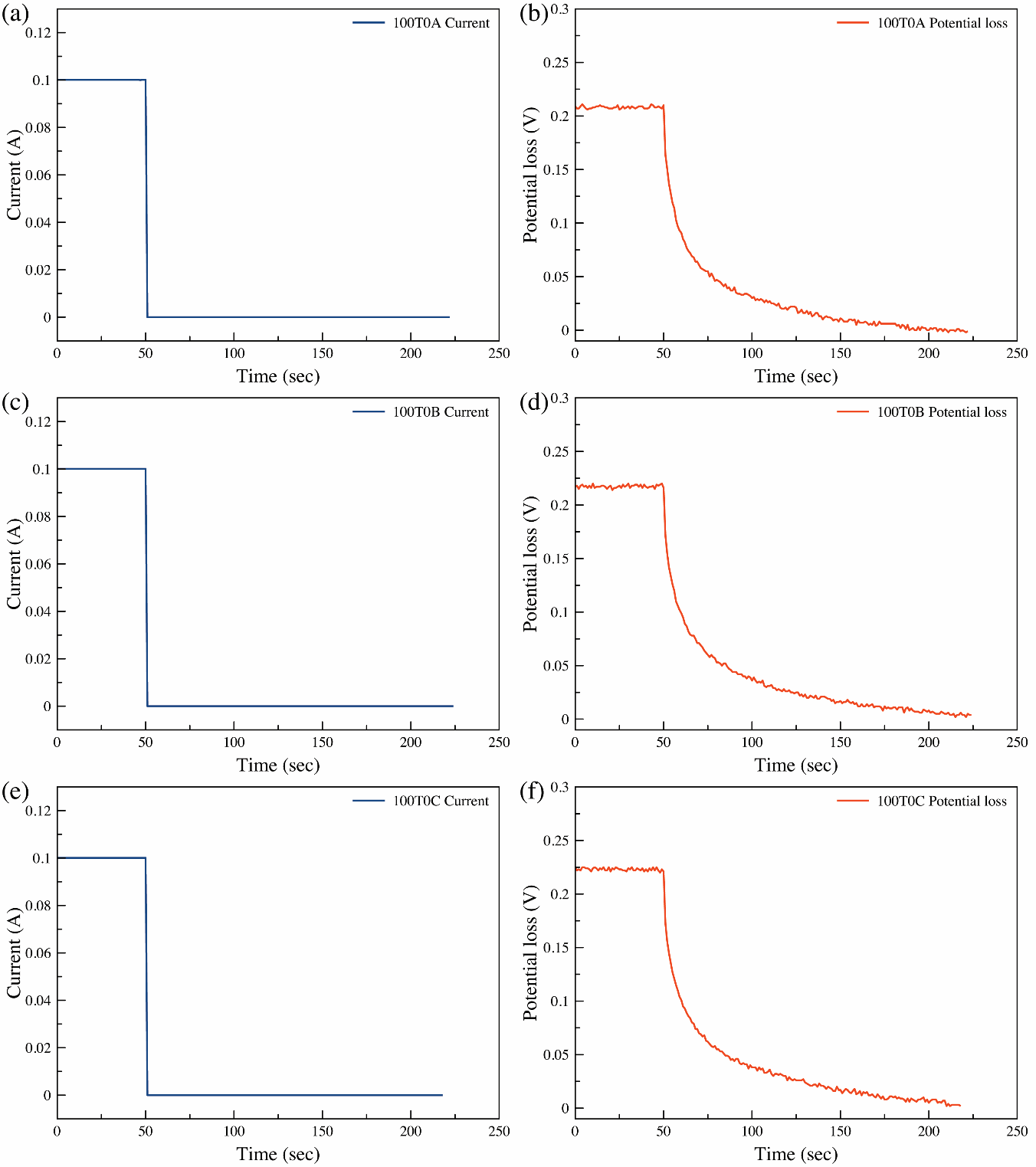


**Figure S4.** Response signals for data 100T0 including (a,c,e) current and (b,d,f) potential loss vs time.


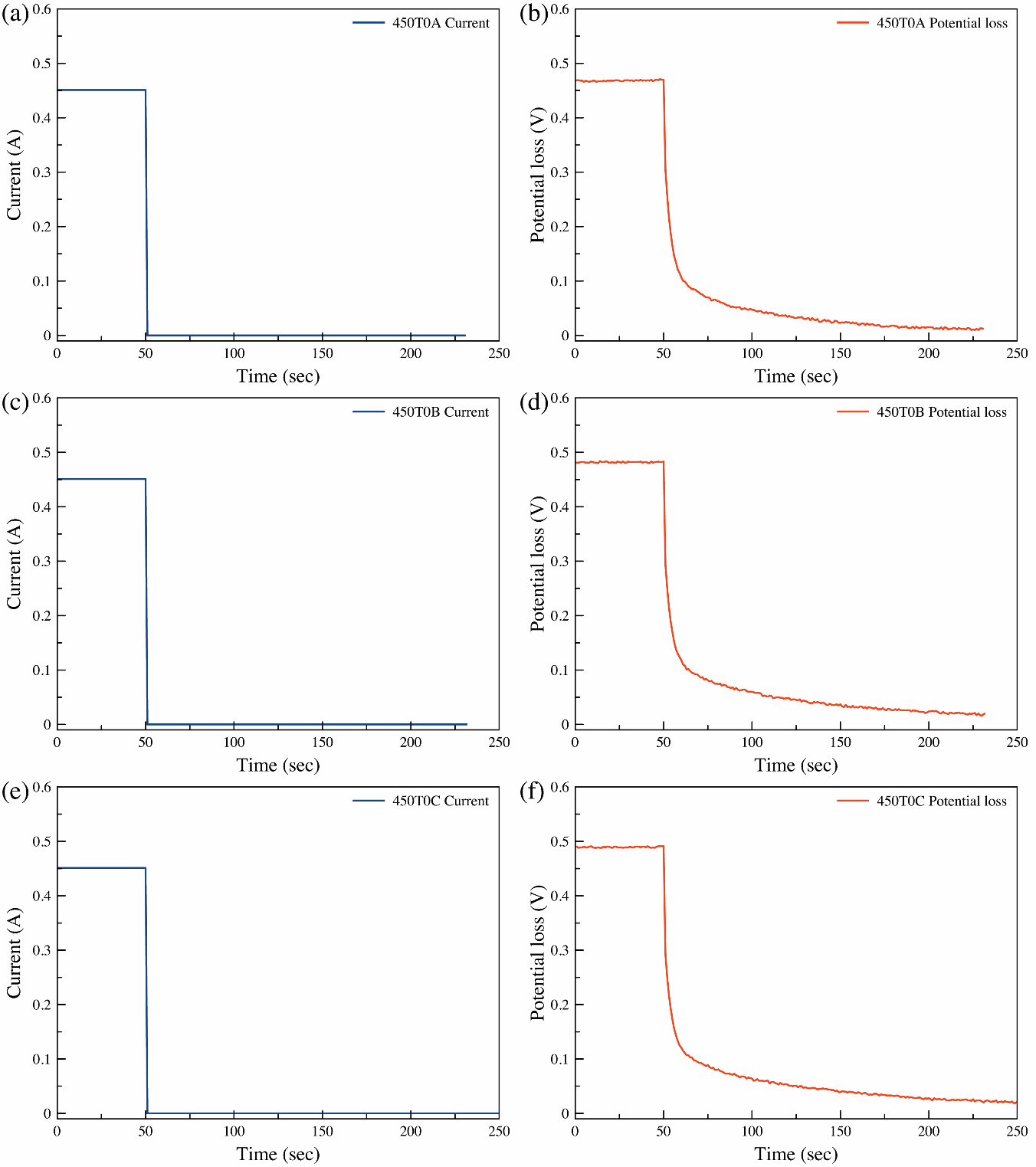


**Figure S5.** Response signals for data 450T0 including (a,c,e) current and (b,d,f) potential loss vs time.


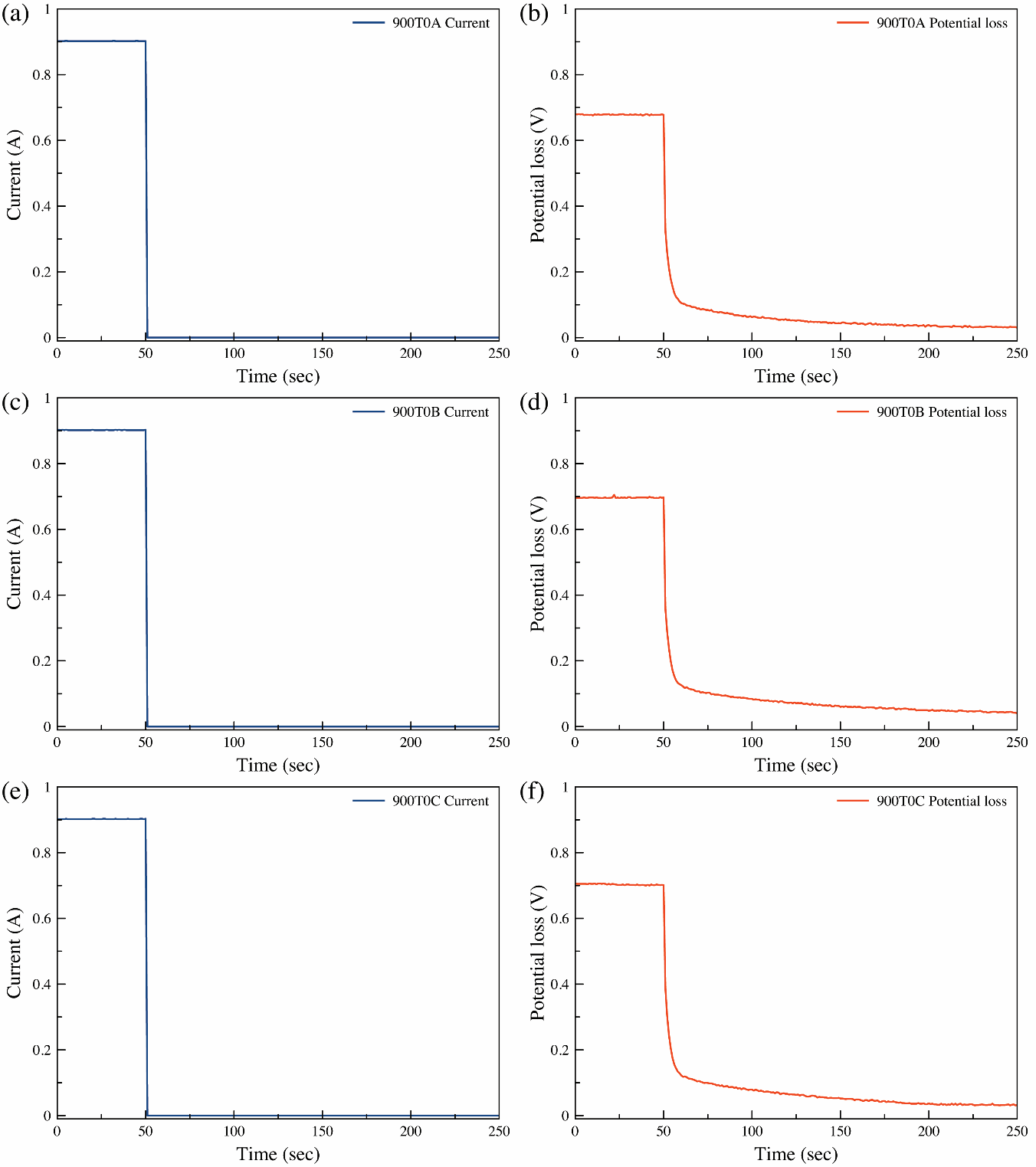


**Figure S6.** Response signals for data 900T0 including (a,c,e) current and (b,d,f) potential loss vs time.


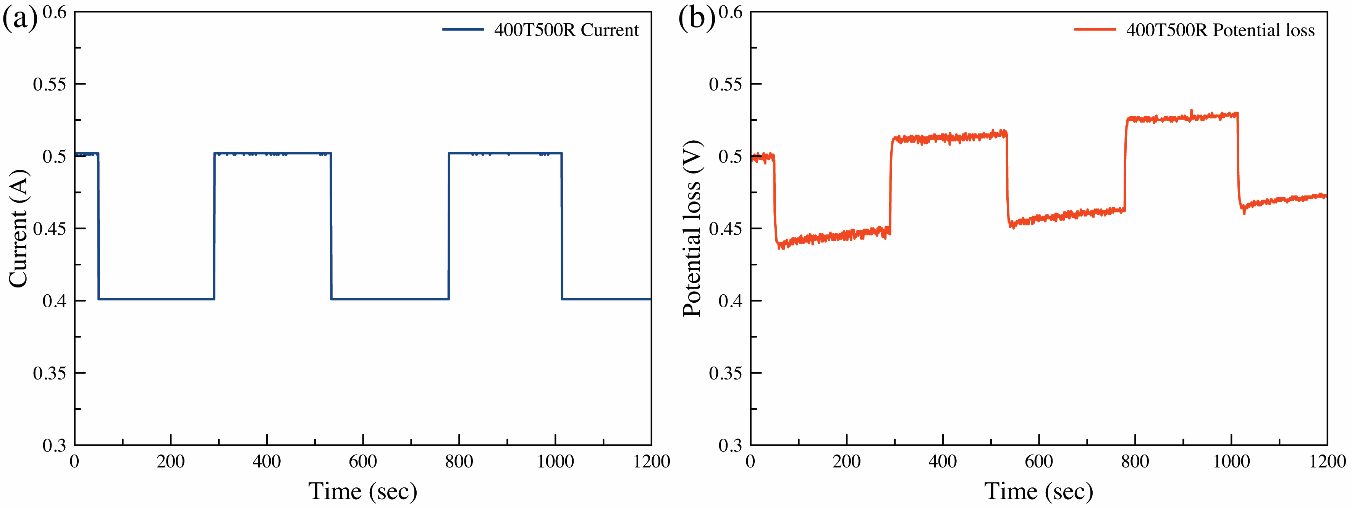


**Figure S7.** Response signals for data 400T500R including (a) current and (b) potential loss vs time.


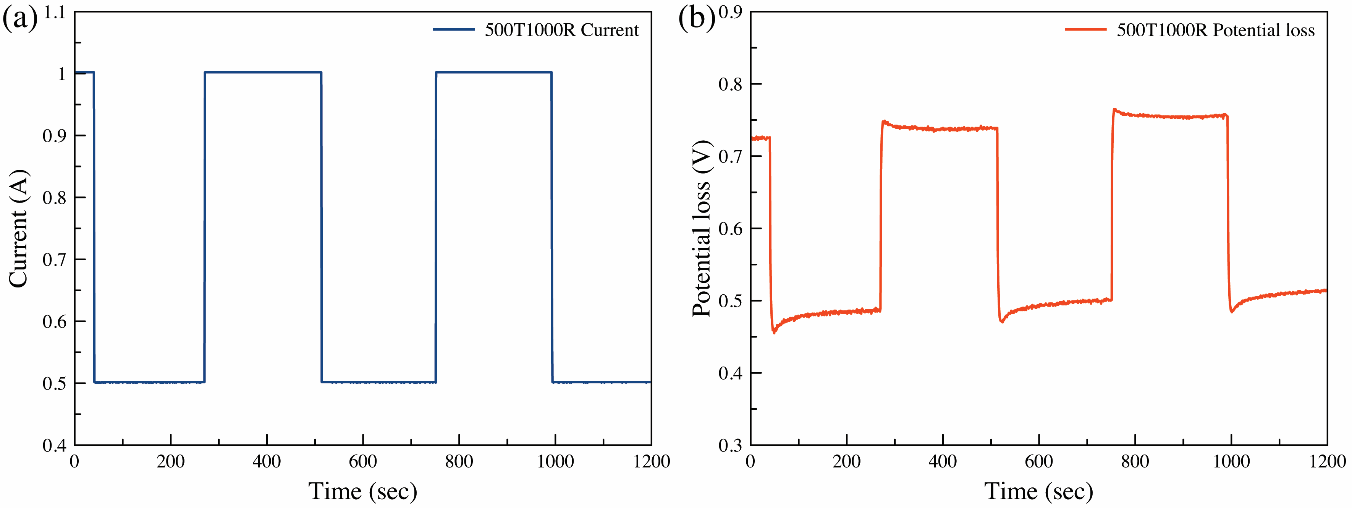


**Figure S8.** Response signals for data 500T1000R including (a) current and (b) potential loss vs time.


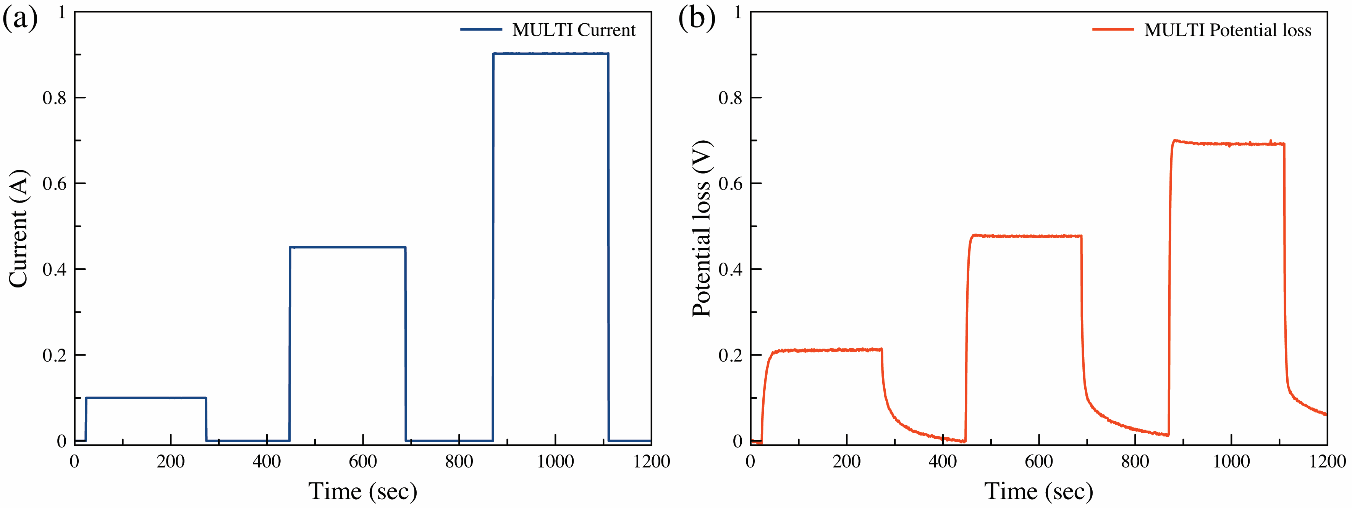


**Figure S9.** Response signals for data MULTI including (a) current and (b) potential loss vs time.


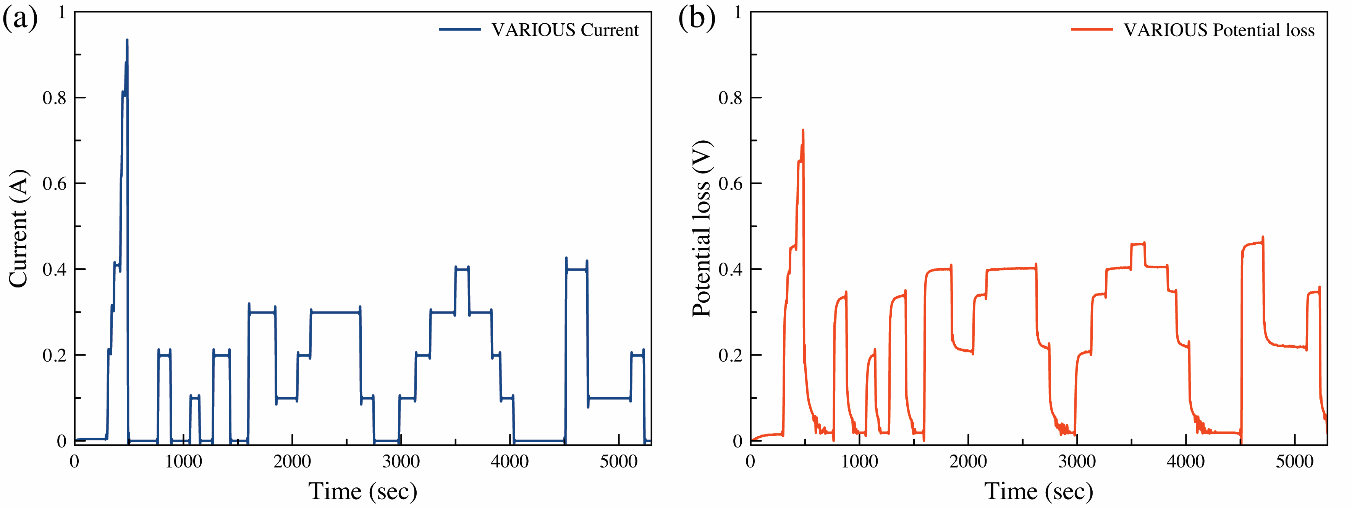


**Figure S10.** Response signals for data VARIOUS including (a) current and (b) potential loss vs time.


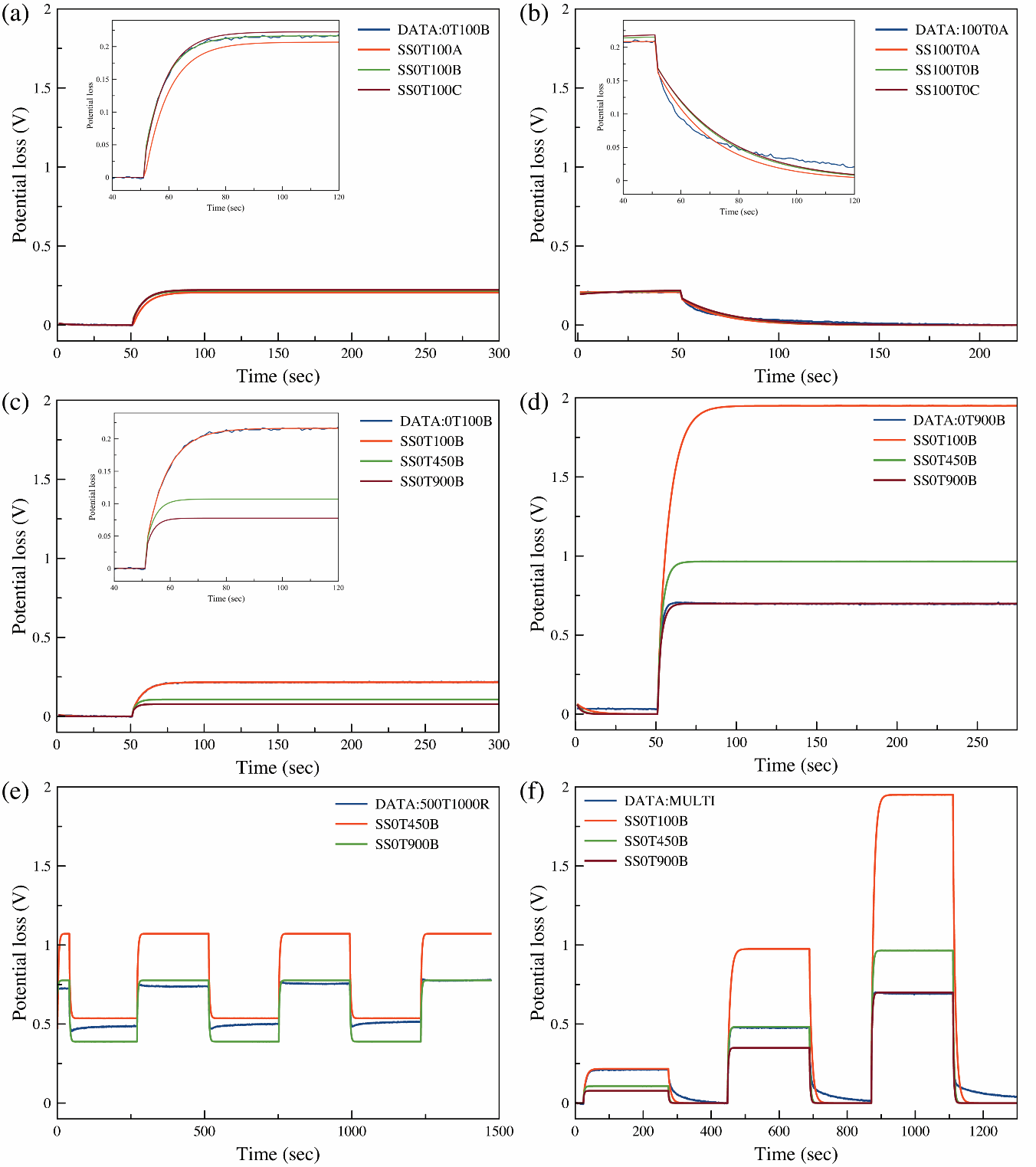


**Figure S11**. Full graphical comparison of response between measured data (blue lines) and predicted data from linear models: a) Matching conditions with current step from 0 to 100 mA b) Matching condition with current step from 100 to 0 mA c) Different conditions with current step from 0 to 100 mA d) Different conditions with current step of 0 to 900 mA e) Repeating current steps between 500 to 1000 mA and f) Multiple current steps from 0 to 100, 450 and 900 mA


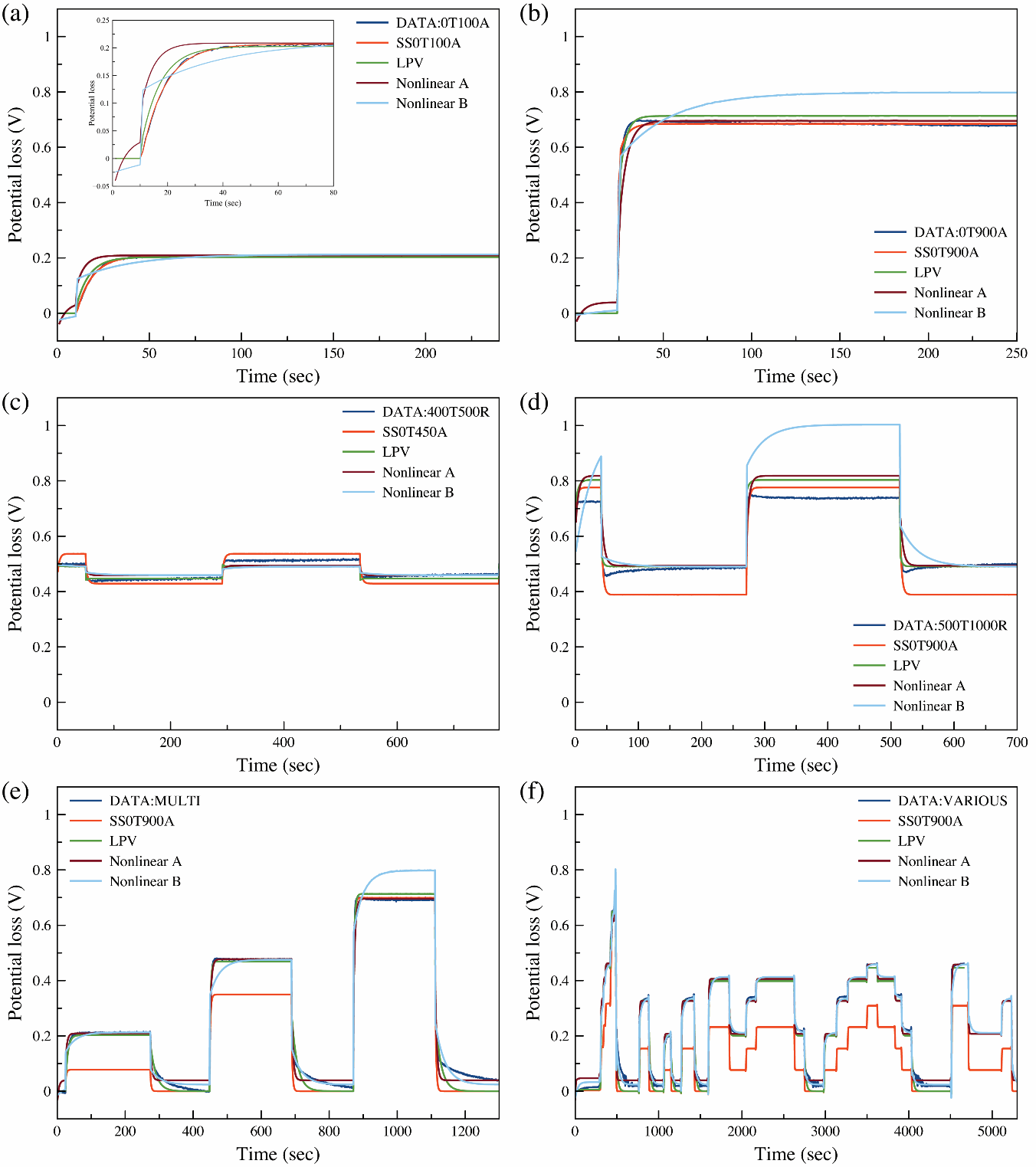


**Figure S12.** Full graphical comparison of response between measured data (dark blue lines), predicted data from linear model (red line), LPV model (green line) and nonlinear models: a) Current step from 0 to 100 mA b) Current step from 0 to 900 mA c) Repeating current steps between 400 to 500 mA d) Repeating current steps between 500 to 1000 mA e) Multiple current steps from 0 to 100, 450 and 900 mA and f) Various current steps

References

1. Lao-atiman, W.; Olaru, S.; Arpornwichanop, A.; Kheawhom, S. Discharge performance and dynamic behavior of refuellable zinc-air battery. *Scientific Data* **2019**, *6*, 168, doi:<https://doi.org/10.1038/s41597-019-0178-3>.
